# An Exotic Species Is the Favorite Prey of a Native Enemy

**DOI:** 10.1371/journal.pone.0024299

**Published:** 2011-09-06

**Authors:** Yiming Li, Zunwei Ke, Supen Wang, Geoffrey R. Smith, Xuan Liu

**Affiliations:** 1 Key Laboratory of Animal Ecology and Conservation Biology, Institute of Zoology, Chinese Academy of Sciences, Beijing, China; 2 Graduate School, Chinese Academy of Sciences, Beijing, China; 3 Department of Biology, Denison University, Granville, Ohio, United States of America; Australian Wildlife Conservancy, Australia

## Abstract

Although native enemies in an exotic species' new range are considered to affect its ability to invade, few studies have evaluated predation pressures from native enemies on exotic species in their new range. The exotic prey naiveté hypothesis (EPNH) states that exotic species may be at a disadvantage because of its naïveté towards native enemies and, therefore, may suffer higher predation pressures from the enemy than native prey species. Corollaries of this hypothesis include the native enemy preferring exotic species over native species and the diet of the enemy being influenced by the abundance of the exotic species. We comprehensively tested this hypothesis using introduced North American bullfrogs (*Lithobates catesbeianus,* referred to as bullfrog), a native red-banded snake (*Dinodon rufozonatum*, the enemy) and four native anuran species in permanent still water bodies as a model system in Daishan, China. We investigated reciprocal recognition between snakes and anuran species (bullfrogs and three common native species) and the diet preference of the snakes for bullfrogs and the three species in laboratory experiments, and the diet preference and bullfrog density in the wild. Bullfrogs are naive to the snakes, but the native anurans are not. However, the snakes can identify bullfrogs as prey, and in fact, prefer bullfrogs over the native anurans in manipulative experiments with and without a control for body size and in the wild, indicating that bullfrogs are subjected to higher predation pressures from the snakes than the native species. The proportion of bullfrogs in the snakes' diet is positively correlated with the abundance of bullfrogs in the wild. Our results provide strong evidence for the EPNH. The results highlight the biological resistance of native enemies to naïve exotic species.

## Introduction

Invasive species can have major negative impacts on the biodiversity and economy in their new range [1], [2], [3], [4], [5]. Understanding the factors shaping the establishment and proliferation of exotic species is, therefore, essential for managing non-indigenous species [6], [7], [8]. The enemy release hypothesis (ERH) supposes that exotic species can become invasive because they escape the effects of natural enemies in their native range [9], [10]. The lack of coevolved enemies and the preference of native enemies for native species due to coevolutionary history promote the competitive advantage of exotic species over native species, which would facilitate the invasions of non-indigenous species. The ‘increased susceptibility’ hypothesis (ISH) (i.e., the ‘new association’ hypothesis) explains why non-indigenous species often fail to establish [6]. This hypothesis posits that invasive species could be subjected to greater enemy effects than source populations or native competitors in invaded regions [6], [11], [12], [13]. As the exotic species have not experienced selection for resistance to the native enemies and perhaps have lost some defenses due to genetic bottlenecks during invasion, they may be unprepared to defend themselves against native enemies. As a result, native enemies may prevent the establishment or spread of non-indigenous species, a form of biotic resistance to invasions of exotic species. The latter is consistent with the predictions of the biotic resistance hypothesis, which assumes that native competitors and enemies can display biotic resistance to biological invasions [2]. Almost all tests of these hypotheses come from the interaction between plants and their enemies [6], [9], [11], [12], [13], [14], [15], [16], [17]. These tests provide mixed support for the ERH, and evidence for the ISH is rare [6], [12], [14], [16]. Few studies have examined predation pressures of native enemies on exotic species in the exotic species' new range [18].

Recent theories emphasize the ability of the naïveté of exotic species or native enemies to influence the establishment and proliferation of exotic species, and the links between naïveté and ERH and ISH in predator-prey systems [6], [8], [13], [19]. Prey naïveté is defined as the lack of effective defenses to enemies due to the absence of an evolutionary history with a given enemy archetype [8]. The lack of effective defenses may include not recognizing an enemy as a predator, using the wrong antipredator response and having an appropriate but ineffective antipredator response [8], [19]. In contrast, enemy naïveté is defined as ineffective predation on prey. An exotic species may enjoy the advantage from the naïveté of a native enemy because the native enemy cannot effectively prey upon the exotic species (referred to as the native enemy naïveté hypothesis)[6], which would facilitate invasion by the exotic species, as predicted by the ERH [9], [10].

Alternatively, an exotic species' naïveté may be a disadvantage because the species would lack effective defenses against the enemy, and therefore, would suffer heavier predation from the enemy than native prey (referred to as the exotic prey naïveté hypothesis) [6]. This scenario could prevent invasions of the non-indigenous species, a result that is consistent with the prediction of the ISH [6], [11]. Native prey may have effective defenses against the native enemy because they have a shared evolutionary history. Because of these defenses, a corollary is that the native enemy may prefer exotic species over native species, everything else being held equal. As a result, the diet of a native enemy may be influenced by the abundance of the exotic species due to the preference of the native enemy for the exotic species. An increase in the abundance of the exotic species may increase the proportion of the exotic species in the diet of the native enemy in the invaded range.

We examine interactions among a native enemy, an exotic species, and native prey species using a native enemy, the red-banded snake (*Dinodon rufozonatum*), an introduced North American bullfrog (*Lithobates catesbeianus,* hereafter referred to as the bullfrog), and native anuran species in permanent still water bodies as a model system, on Daishan Island of the Zhoushan Archipelago, China. The red-banded snake is widely distributed in South and East Asia, including most areas of China (except Tibet, Ningxia, Qinghai and Xinjiang), the Russian “Far East”, North Korea, South Korea, Southern Japan, Vietnam and Laos [20], [21]. The snake is a generalist native predator, feeding mainly on different frogs (including bullfrogs), fishes, small reptiles and small mammals [20], [22]. The bullfrog is native to eastern North America [23], and is considered one of the 100 worst invasive species in the world [24] and has been widely introduced into over 40 countries [23]. Bullfrogs can swallow any prey that are smaller than the bullfrog's mouth, and have been responsible for the decline or extinction of some native amphibians in bullfrog-invaded areas through predation, as well as through competition and the spread of disease [25], [26], [27], [28], [29]. Both the red-banded snake and the bullfrog depend on freshwater habitats. Freshwater systems are known to exhibit high heterogeneity in predation regimes [8], which limit biotic interchanges and promote the naiveté of prey and enemies in the systems. We hypothesized that if the bullfrog were naïve toward the red-banded snake, the bullfrog would suffer higher predation pressures from the snake than native anuran species, and the proportion of bullfrogs in the snake's diet would be positively correlated with the abundance of bullfrogs in the wild. In contrast, if the snake were naïve to the bullfrog, the bullfrog would be subjected to lower predation pressures from the snake than native anuran species.

We first determined the naïveté of bullfrogs and red-banded snakes via olfactory communication (or chemical detection) experiments for reciprocal recognition between the snake predator and amphibian prey. Failing to recognize an exotic prey or a native enemy is considered the most damaging form of naïveté for both the enemy and the prey, respectively, because this form of naïveté can reduce the enemy's predation on the prey or the prey's defenses against the enemy [8], [19]. Olfactory communication plays a key role in reciprocal recognition between snake predators and amphibian prey [30]. Many snake genera use chemical cues to detect and discriminate prey [31], [32], [33], [34]. Both amphibian larvae and post-metamorphic individuals (including bullfrogs) use chemical cues to assess predation risk [30], [35], [36], [37], [38], [39]. Recognition of invasive predators by native prey based on chemical cues is well documented, but studies have rarely considered the behavioral responses of exotic species to the chemical cues of native enemies [40]. Native prey species can show pronounced avoidance responses to chemical cues associated with native predators [41], [42], [43], [44], [45]. Some native species show little or no response to chemical cues of novel predators [36], [39], [46], [47], whereas others can recognize invasive predators or are able to learn or evolve the ability to avoid chemical cues of invasive predators [35], [45], [48], [49], [50].

We performed chemical detection experiments for reciprocal recognition in two parts. In the snake tongue flick experiment, we measured the response of red-banded snakes toward native anurans and bullfrogs, and in the anuran chemical cue avoidance experiment, we measured the response of native anurans and bullfrogs to the chemical cues of the snake. We then compared predation pressures of red-banded snakes on bullfrogs and native anuran species by investigating the diet preference of the snake for bullfrog versus native amphibian species in laboratory and field experiments to link the number of bullfrog and native amphibian species in the snake diet (killed by the snake) to their abundance. Finally, we examined the relationships between the proportion of bullfrog and native amphibian species in the snake's diet and their abundances in the wild.

## Methods

### Ethics Statement

This study was approved and supervised by the Animal Care and Use Committee of Institute of Zoology, the Chinese Academy of Sciences (Project No. 2008/73). Permits for animal collection were obtained from the Daishan Agriculture and Forestry Office. All staff, fellows and students received appropriate training before performing animal studies.

### Study Area

Our study was conducted on Daishan Island (30°14′–30°20′ N and 122°05′–122°14′ E) of the Zhoushan Archipelago in Zhejiang Province, China (Supporting Information S1). Daishan is the second largest island (104 km^2^) in the archipelago [51], and its topography consists of 60% hills (total area) and 40% plains. The highest peak on the island is 257 m. Rivers are relatively rare. Permanent still water bodies (PSWBs) include ponds and reservoirs, which are located at the foot of hills, and provide the main source of freshwater for human needs and agricultural irrigation. The island is in a highly seasonal sub-tropical ocean monsoon zone. Mean temperature is 5.3 °C in January and 27.3 °C in August. Annual precipitation is about 1000 mm. The natural vegetation is dominated by a sub-tropical evergreen broadleaf forest. There are eight native amphibian species and sixteen indigenous snake species on the island [52], [53]. Human population density is approximately 1000 persons per km^2^.

### Study system

Bullfrogs escaped from bullfrog farms or released by human activities had invaded most PSWBs on Daishan Island by the mid 1990s [54], [55]. The red-banded snake is the only snake species around these water bodies. The snake is mid-sized (Supporting Information S2) and lives around ponds, reservoirs, rivers, streams, rice fields, bogs and ditches of dry land [20]. They breed between May and August on the island. Rice frogs (*Fejervarya limnocharis*), pond frogs (*Rana. nigromaculata*) and toads (*Bufo bufo*), are the most common native anuran species occurring in PSWDs on Daishan Island [55], accounting for over 95% of the total native amphibian abundance in these water bodies. Other native amphibians include Japanese frogs (*R. japonica*), which are occasionally found in PSWDs. All of the snakes, bullfrogs and the native anuran species are nocturnal [20], [21].

### Chemical detection experiments between red-banded snakes and anurans

#### The capture and maintenance of snakes and anurans

We used red-banded snakes, bullfrogs and three common native anurans (pond frogs, rice frogs and toads) in the experiments. Because experienced snakes may behave differently from inexperienced ones when they encounter prey [35], [36], we captured snakes in rice fields, ponds and reservoirs by hand or net, in locations where bullfrogs have not invaded yet (no tadpoles, post-metamorphic individuals, eggs or calls of bullfrogs were detected during 3 consecutive years of field investigations) (Supporting Information S3). We captured bullfrogs and the three native anuran species from ponds or reservoirs where we had detected red-banded snakes.

#### Snake tongue flick experiment

We conducted the experiment in a terrarium (150 cm×75 cm×70 cm) divided by a removable opaque baffle in the middle (Supporting Information S4) from 4–13 October 2008. We first gently rubbed the body of an individual frog or toad onto the whole outside surface of a steel strainer (15-cm diameter, 7 cm deep, mesh size of 1 mm) [56], and then placed the individual under the strainer, which was then taped to the floor at the center of one side of the terrarium. We then placed a snake at the center of the other side of the terrarium. The snake was allowed to acclimate to the terrarium for approximately 15 min, after which the baffle was gently raised to allow the snake access to the whole terrarium. When the snake's head entered the part of the terrarium containing the strainer, we recorded their behaviors for 10 min using a video camera. We calculated the time spent by the snake flicking its tongue (hereafter time scores, TS) toward the strainer and counted the number of tongue flicks directly contacting the strainer (hereafter tongue flick scores, TFS). TS is defined as the amount of time spent flicking the tongue in 10 min by the snake toward a prey and TFS as the number of tongue flicks in 10 min. Both indices have been widely used to measure snakes' preference for prey [56], [57], [58]. Higher TFS and TS may represent a greater preference of the snake to its prey. The terrarium was cleaned with soap and bleach to remove all odor cues, rinsed, and dried between trials. To remove effects of order of anuran species tested and prior experience on the subsequent behavior of snakes [36], [59], [60], we systematically balanced the order of presentation of anurans among the species [56]. In total, 100 frogs (25 individuals from each anuran species), 25 control containers, and 25 snakes were used in the experiment. We used each individual anuran only once, and each snake was subjected to 5 trials (one trial for each of three native anurans, one trial for bullfrogs, and one for the control containers).

#### Chemical cue avoidance of the anurans to snakes

Our methods followed the protocols used by [36], [61]. Prior to the behavioral trials, we rinsed and sun-dried paper towels, and then placed them on the floor of bins into which snakes were placed for 36 h. Snakes were fasted for a minimum of 5 d before being placed in the bins. We removed any fecal material that accumulated on the paper towels. We built 20 chambers (24 cm×45 cm×45 cm). A fume hood was placed over these chambers to prevent cross-contamination of predator cues. Two paper towels were placed on the floor of each chamber, one on each side of the chamber with a 2 cm gap along the center line of the chamber to avoid diffusion of chemical cues. For the snake treatment, a paper towel with snake chemical cue was placed on one side and a clean towel on the other side. For the control treatment, both sides of the chamber received a clean paper towel. An individual anuran was placed on the central line of the chamber with its head parallel to the central line. We recorded the position of the frog or toad in the chamber every two minutes for 2 hours using a DV camera [35], [36]. We defined the position of the individual as being on one side when the majority of the body was on that side. Five chambers were placed on a rotatable desk, and 2 rotatable desks were used. Every 30 min, we very slowly rotated the chamber 180° using the rotatable desk. We misted the paper towels with de-chlorinated water every 20 minutes to minimize the potential for dehydration during trials. The experiment was conducted from 4 to 16 October 2008 in a dark room at room temperature around 28°C and under fluorescent lighting. In between trials, we cleaned (soap and bleach), rinsed, and dried the chambers to remove all odor cues. For each anuran species, we used 20 individuals for the snake treatment and 20 for the control treatment. We defined the chemical cue avoidance reaction as the proportion of time that an individual spent on the control side of a chamber.

### The diet preference experiments of red-banded snakes for anurans

The experiments were conducted in a group of abandoned artificial ponds for aquaculture on Taohua Island, 40 km from Daishan Island (Supporting Information S5). Nine artificial ponds (13.5 m in length × 6.5 m in width × 1.4 m in depth) and nine snakes (one snake in each pond) were randomly assigned to three treatments: A) a control group of anurans without bullfrogs, B) bullfrogs (2 individuals) and native anurans, and C) bullfrogs (2 individuals) and native anurans controlling for body size (Supporting Information S6). The native anurans in each treatment included 2 rice frogs, 2 pond frogs and 2 toads. Each pond was a well constructed of brick and concrete around a foundation of soil. The foundation had a small slope. Fresh water was drawn from a near reservoir into each pond so as to cover two-thirds of the floor (30 cm maximum water depth). The water level in each pond was maintained with a water supply and drainage system throughout the experiment. Each pond was covered with a sunshade screen to protect the subjects from birds of prey. Some bricks and stones were placed on the pond floors as shelters for the animals. In treatments A and B, bullfrogs or native anurans were randomly sampled from animals captured from the wild. As great differences in body size exist among anuran species, which might affect the preference of red-banded snake (Supporting Information S2), treatment C was designed to control for potential effects of body size. In doing so, individuals with no differences in body size (in either SVL or body mass) for anuran species (sub-adults for bullfrogs) were chosen from the individuals captured (Supporting Information S6).

We captured and kept animals in the same way as during the recognition experiments described above. Snakes were fasted for at least 5 days and anurans were fasted for 48 hours prior to the trials. We only used females and juveniles in the experiment. We marked each animal with a visible implant elastomer tag [62]. We placed the red banded snakes, bullfrogs, and each of the native anuran species into separate ponds for one day to habituate them to their new environment. We then randomly assigned animals to ponds according to the treatments. We checked to see whether any anuran individuals were hunted by the snake or bullfrogs in each pond every night (1930 h–2330 h). Observers with a 12 volt DC lamp entered the pond to carefully determine whether any anurans were lost from a pond. When native individuals were missing, we would induce the snakes to regurgitate to recover prey items from the stomach [63] or flush the stomach contents of bullfrogs with water [64]. We only induced snakes to regurgitate when bullfrogs were missing. We then replaced the consumed individual with a new one (of the same species and with a similar body size) into the pond to keep a constant availability of bullfrogs or native anurans. Bullfrogs whose stomachs were flushed were also replaced with new ones to remove the effects of flushing the stomach on the anti-predator behaviors of the bullfrogs to the snake. Stomach contents were identified to species. Prey items in the stomach contents were weighed to the nearest 0.1 g. The experiment lasted for 21 days from 24 September to 15 October 2008.

### Field survey

#### The diet of red-banded snakes

We investigated the diet of red-banded snakes in PSWDs from 16 June to 26 September 2009 on Daishan Island. We carefully searched for red-banded snakes in water-fluctuation belts along the accessible banks of each water body at night (1930 h–2400 h) with an electric torch (12 volt DC lamp). We captured the snakes by hand, and recorded the location of each snake captured by GPS. We searched each water body twice. We first surveyed a water body for three consecutive nights. One month later, we searched the water bodies again. We induced snakes to regurgitate at the time of capture to recover prey items from the stomach [63]. Stomach contents were identified to species when possible, and contents were counted. Prey items were weighed to the nearest 0.1 g. We measured snout-vent length (to the nearest 0.02 mm) and body mass (to the nearest 0.1 g) of the snakes, and marked the snakes using scale clipping [63]. The diet of the red-banded snake in a PSWB was defined by summing diet of the snakes in the PSWB. Snakes were released at the site where they were captured.

#### Anuran abundance in PSWDs

After the snake survey each night, we investigated the abundance of anurans using the line transect method [65], [66]. We fixed transects (2 m×10 m) along the bank with half the width of the transect (1 m) in the water and half on the bank. The shoreline of a water body (accessible parts) was divided into 5 segments of equal length. We randomly located a line transect in a segment and sampled transects with an electric torch (12 volt DC lamp). We carefully counted frogs and toads encountered along transects [66]. We located transects in a different randomly chosen position each night. We also measured the maximum depth and surface area of each water body [66]. We calculated the abundance of each anuran species in a water body as the sum of the number of individuals of the species in all line transects on six nights.

### Statistical analyses

For the snake chemical cue avoidance experiment, we used an arcsine-square root transformation to normalize the data for each anuran individual; then, we tested whether anuran species randomly used the paper towel with snake chemical cues or the clean paper towel (i.e., 50∶50) using a one-sample *t*-test (Murray et al. 2004). For the snake tongue flick experiment, TFS and TS for each anuran individual were normalized using a ln (x+1) transformation. We examined differences in TFS and TS among prey treatments using one-way ANOVAs. Levene's test of homogeneity of variances showed that the variance was not equal across groups; therefore, a post hoc Tamhane's test of pair-wise contrasts was used to detect differences between prey treatments.

We used Jacobs' index to represent the preference of red-banded snakes for bullfrog and native anurans [67], [68]. 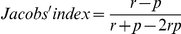
where *r* is proportion of an anuran species in the diet by prey number; *p* is proportion of the anuran species available in the environment. This index was used because it was relatively independent of sizes of prey samples and the relative abundances of prey species in the environment [67]. The index has a range of −1 to +1, with −1 indicating maximum avoidance, +1 indicating maximum preference and 0 indicating random selection. We calculated the mean Jacobs' index for each prey species across artificial ponds in each treatment, and across PSWBs in bullfrog-invaded sites and non-invaded sites.

For the experiments, we tested if the mean Jacobs'index in a treatment indicated significant preference or avoidance using *t*-tests against a mean of 0 [68]. Then, we tested differences in mean Jacobs'indexes among anuran species using a one-way ANOVA. We performed multiple comparisons using the LSD test. For the field survey, we examined preference or avoidance of the mean Jacobs'index value against a mean of 0 using *t*-tests if the assumption of normality were met, and using the sign test if the assumption of normality was not met [68]. We examined differences in mean Jacobs'indexes among prey species using Kruskal-Wallis test. We then performed multiple comparisons using Mann-Whitney *U* tests. The Bonferroni adjustment for multiple comparisons was used to maintain a consistent overall error rate by reducing α value (0.05) to *α/m* (m = number of comparisons, *α/m* = 0.0083 if number of prey species = 4; 0.005 if number of prey species = 5).

We determined the relationship between the averaged proportions (arcsin-transformed) of an anuran species (in number) in the diet of red-banded snakes for each species in a water body where snakes were found with prey in their stomachs and the anuran species' density in the water body (number, ln(1+x) transformed) using a Spearman rank correlation test.

## Results

### Chemical detection between red-banded snakes and anurans

In the chemical cue avoidance experiments, the proportion of time spent did not differ between both sides of chambers for each anuran species in the control trials, suggesting that all four of the anurans used the substrates randomly (Table 1). Bullfrogs and toads showed no difference in percent time spent between the paper towel with snake chemical cue and the clean paper towel (Table 1). However, rice frogs and pond frogs displayed a non-random use of the two substrates, spending significantly more time on the clean paper towel side.

**Table 1:** Use of control substrates (Mean±SD) by four amphibian prey species in a chemical cue avoidance experiment with red-banded snakes.

Species	Treatments	Proportion	t-value	P-value
*R. catesbeiana*	Control	0.48±0.23	0.41	0.69
*R. limnocharis*	Control	0.51±0.1	0.42	0.68
*R. nigromaculata*	Control	0.49±0.21	0.24	0.81
*Bufo bufo*	Control	0.51±.16	0.29	0.78
*R. catesbeiana*	*D. rufozonatum*	0.52±0.22	0.42	0.68
*R. limnocharis*	*D. rufozonatum*	0.57±0.11	2.979	0.008
*R. nigromaculata*	*D. rufozonatum*	0.57±0.14	2.25	0.036
*Bufo bufo*	*D. rufozonatum*	0.54±0.18	0.93	0.363

Each trial involved 20 prey animals, and analyses were performed using one-sample t-tests, which evaluated the hypothesis of random use (i.e., 50: 50 use) of substrates. Analyses were performed on transformed proportions.

**Figure 1:**
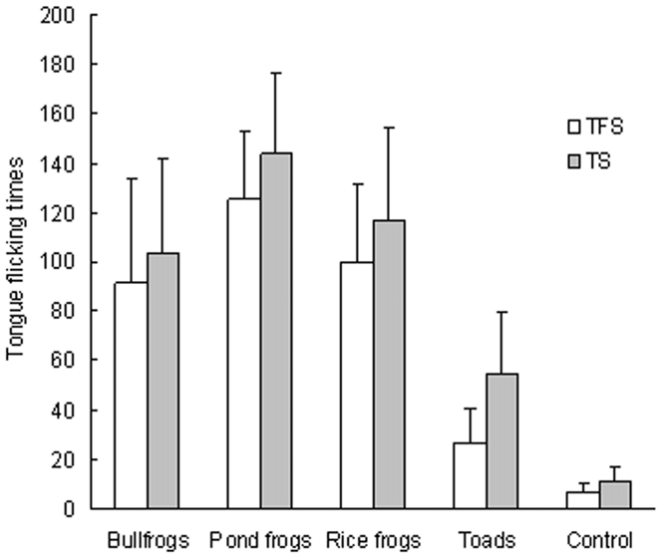
Mean tongue flick scores (± SE) and mean flick time (± SE) in 10 minutes by red-banded snakes towards different anuran prey species.

In the snake tongue flick experiments, snakes showed differences in TFS and TS toward anuran species and the control treatment (One-way ANOVA, *F_4,124_* = 169.90, *p*<0.001 for TFS; *F_4,124_* = 166.42, *p*<0.001 for TS) (Fig 1). Snakes performed more TFS and TS toward pond frogs than toward bullfrogs, toads, and control treatments (Tamhane's test, TFS: *p* = 0.015 for bullfrogs, *p*<0.001 for toads and control; TS: *p* = 0.01 for bullfrogs, *p*<0.001 for toad and control). Furthermore, the snakes displayed more TFS and TS toward rice frogs and bullfrogs than toward toads and control treatments (*p*<0.001 for both TFS and TS) and more TFS and TS towards toads than control treatments (*p*<0.001 for both TFS and TS).

### The diet preference of red-banded snake in artificial ponds

**Figure 2:**
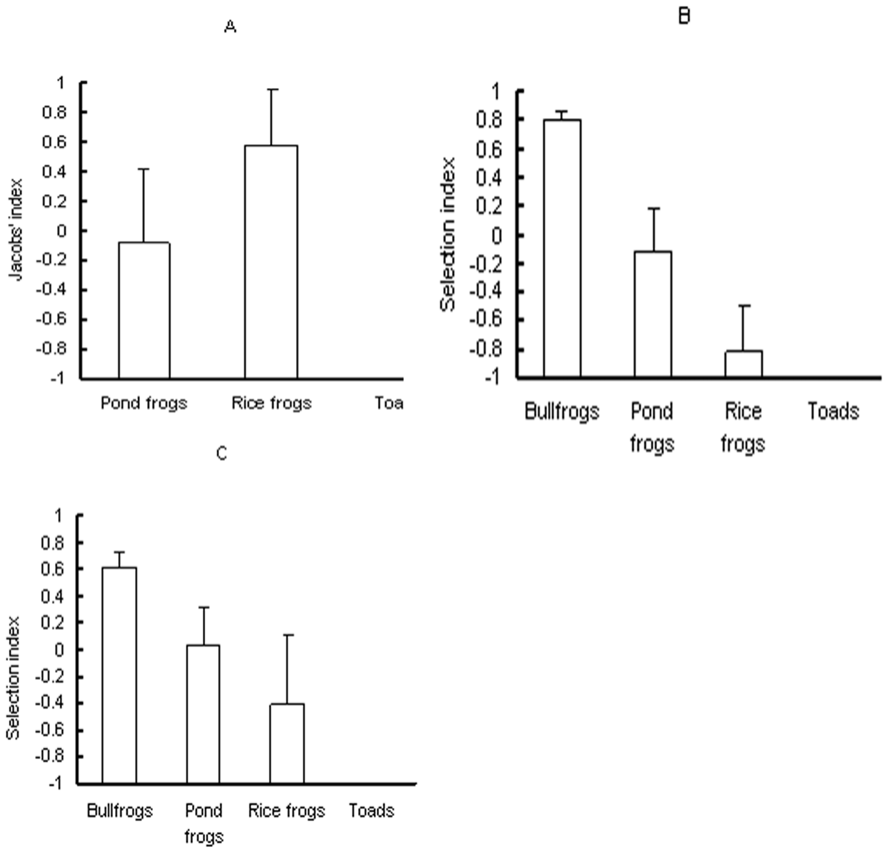
The Jacobs' index (± SD) of red-banded snakes for bullfrogs vs. three native anuran species in artificial ponds. A. control treatment; B. bullfrogs and native anurans with random body size; C. bullfrogs and native anurans with similar body size.

In three control ponds, the snakes preyed upon a total of 17 frogs (total wet mass: 303.3 g). Pond frogs and rice frogs were the main food items of the snakes, accounting for 29.41% and 70.59% of the snake diet in number, and 60.24% and 39.76% of the wet mass of the diet, respectively. The Jacobs'index for toads in all three ponds was −1 (Figure 2A), indicating maximum avoidance for the snakes (*t* test could not be performed due to the standard deviation = 0). Snakes did not show a preference for or avoidance of either of the two native frog species (*t* = −0.294, *p* = 0.796 for pond frogs; *t* = 2.654, *p* = 0.117 for rice frogs). A one-way ANOVA showed that Jacobs'indexes differed among the three species (*F_3,8_* = 14.01, *p* = 0.005). The Jacobs'index of pond frogs and rice frogs was higher than that of toads (LSD test, *p* = 0.023 for pond frogs; *p* = 0.002 for rice frogs).

In the ponds containing anuran species with random body size, the snakes consumed 20 frogs (total wet mass: 1001.5 g). Bullfrogs were the main food item of the snakes, comprising 75% of the diet in number and 89.7% of the wet mass, whereas pond frogs and rice frogs accounted for 20% and 5% of the diet in number, and 9.14% and 1.16% in wet mass, respectively. The snakes preferred bullfrogs (*t* = 19.392, *p*<0.001) but avoided rice frogs (*t* = −4.5, *p* = 0.046) and toads (the Jacobs'index for toads in all three ponds was still −1) (Figure 2B). The Jacobs'indexes differed among the four anuran species (one-way ANOVA, *F_3,8_* = 37.621, *p*<0.001). The snakes preferred bullfrogs more than any of the native anuran species (*p* = 0.001 for pond frogs; *p*<0.001 for rice frogs and toads). Furthermore, the Jacobs'index of pond frogs was higher than those of rice frogs (*p* = 0.006) and toads (*p* = 0.002).

In the treatment with anuran species of similar body size, snakes consumed 15 frogs (total wet mass: 238.5 g). Bullfrogs were the main food item for the snakes, comprising 60% of snake diets in number and 59.66% of the wet mass. Pond frogs and rice frogs accounted for 26.67% and 13.38% of snake diet in number, and 28.55% and 11.78% in wet mass, respectively. The snakes still preferred bullfrogs (*t* = 9.838, *p* = 0.01) but avoided preying on toads (the Jacobs'index for toads in all three ponds was −1) (Figure 2C). Differences in Jacobs'indexes were detected among anuran species (*F_3,8_* = 15.277, *p*<0.001). Again, bullfrogs were the most favored food item among the species (*p* = 0.045 for pond frogs; *p* = 0.003 for rice frogs; *p*<0.001 for toads). The snakes also preferred pond frogs and rice frogs more than toads (*p* = 0.003 for pond frogs; *p* = 0.046 for rice frogs).

### The diet preference of red-banded snakes in PSWDs

We captured 240 snakes in 103 PSWDs on Daishan Island, including 60 snakes (18 snakes with prey in their stomachs) in 19 non-invaded water bodies and 180 snakes (64 snakes with prey in their stomachs) in 84 bullfrog-invaded water bodies. There was no difference in the proportion of snakes without prey in their stomach in non-invaded water bodies and invaded water bodies (Chi-squared test, *X* ^2^ = 0.716, *df* = 1, *p* = 0.398). The snakes preyed on 197 prey items weighing 4007.6g in wet mass, including 193 anurans (97.97%) weighing 3919.9 g (97.81%), 3 fish (1.52%) weighing 73 g (91.82%), and one lizard (0.51%) weighing 14.7 g (0.37%).

**Figure 3:**
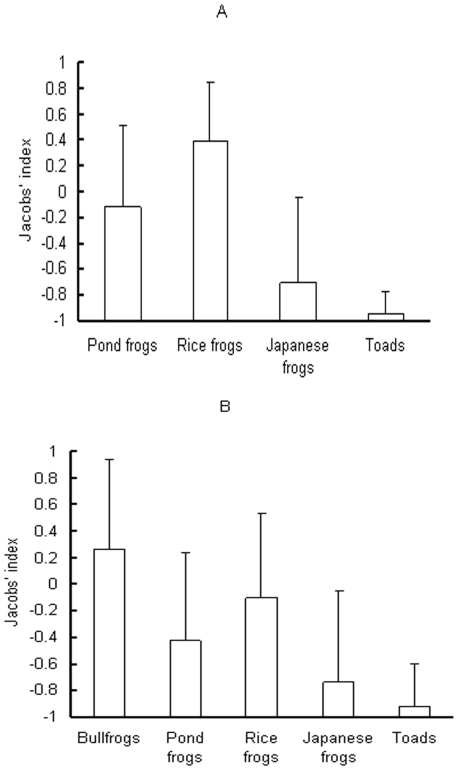
The Jacobs' index (± SD) of red-banded snakes for anuran species in permanent still water bodies on Daishan Island, China. A. in 14 non-bullfrog invaded water bodies where snakes had amphibian prey in their stomachs. B. in 44 bullfrog-invaded water bodies where snakes had amphibian prey in their stomachs.

Pond frogs and rice frogs were important prey items for the snakes in non-invaded water bodies, accounting for 57.89% ( = 22/38) and 34.21% (13/38) of amphibian prey in number and 37.47% (170.6/455.3g) and 54.95% (250.2/455.3) in wet mass, respectively. Other food items included toads (2.63% = 1/38 in number, 3.43% = 15.6/455.3 in wet mass) and Japanese frogs (5.26% = 2/38 in number, 4.15% = 18.9/455.3 in wet mass). The Jacobs'index of rice frogs was higher (*t* = 3.142, *df* = 13, *p* = 0.008) but the Jacobs'index of Japanese frogs and toads was lower (Sign test, *N* = 12, *S_-_* = 10, *p* = 0.019 for Japanese frogs; *N* = 14, *S_-_* = 14, *p*<0.001 for toads) than a mean of 0 (Figure 3A), suggesting that the snakes preferred rice frogs and avoided preying upon Japanese frogs and toads. Jacobs'indexes differed among the four native anuran species (Kruskal-Wallis test, *X^2^* = 27.867, *df* = 3, *p*<0.001). The snakes preferred rice frogs more than Japanese frogs (Mann-Whitney *U* test, *U* = 19, *N* = 26, *p*<0.001<0.0083) and toads (*U* = 0, *N* = 28, p<0.001), and pond frogs more than toads (*U* = 31, *N* = 28, *p*<0.001) (Figure 3A).

In invaded sites, snakes consumed 148 anurans weighing 3349.5 g in wet mass. Bullfrogs were a main food item, comprising 52.0% of the snake diet in number and 69.8% of the wet mass. Pond frogs accounted for 16.2% of the snake diet in number and 18.1% of the wet mass, rice frogs 28.4% in number and 10% in wet mass, Japanese frogs 1.4% in number and 0.5% in wet mass, and toads 2% in number and 1.6% in wet mass. Bullfrogs were the preferred prey of the snakes (*t* = 2.684, *df* = 43, *p* 0.01), but native anuran species, except rice frogs, were not preferred (Sign test, *N* = 44, *S_-_* = 28, *Z*<1.96, *p* = 0.025 for pond frogs; *N* = 22, *S_-_* = 19, *Z*<−3.624, *p*<0.001 for Japanese frogs; *N* = 44, *S_-_* = 43, *Z*<−6.482, *p*<0.001 for toads) (Figure 3B). A Kruskal Wallis test revealed differences in Jacobs'indexes among prey species (*X^2^* = 68.91, *N* = 198, *p*<0.001). The Jacobs'index of bullfrogs was higher than those of four native anuran species (*U* = 584, *N* = 88, *p* = 0.001<0.005 for rice frogs; *U* = 441, *N* = 88, *p*<0.001 for pond frogs; *U* = 196, *N* = 66, *p*<0.001 for Japanese frogs; *U* = 214, *N* = 88, *p*<0.001 for toads). Moreover, the snakes preferred rice frogs to Japanese frogs (*U* = 240, *N* = 66, *p*<0.001) and toads (*U* = 320.5, *N* = 88, *p*<0.001), and pond frogs to toads (*U* = 590, *N* = 88, *p*<0.001) (Figure 3B).

**Figure 4:**
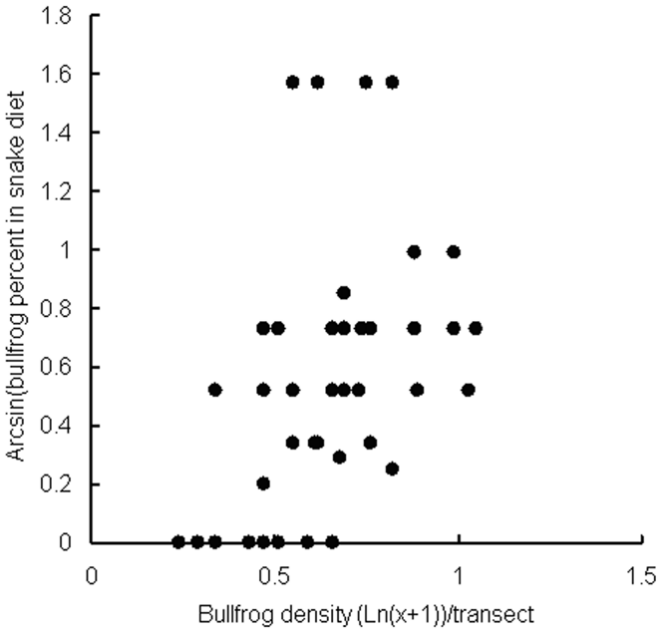
The relationship between the proportion of bullfrogs in the diet of red-banded snakes and bullfrog density in permanent still water bodies where snakes with amphibian prey in their stomachs were captured on Daishan Island, China.

The proportion of bullfrogs in snake diets was positively correlated with the density of bullfrogs in the water bodies (Figure 4; Spearman's rank correlation, *r* = 0.477, *N* = 44, *p* = 0.001). In addition, the percent of rice frogs in the snake diet was also positively correlated with their density (*r* = 0.309, *p* = 0.041).

## Discussion

The results of this study support the exotic prey naïveté hypothesis. In the chemical cue avoidance experiments, there was no difference in percent time spent by bullfrogs on the paper towel with the snake chemical cue and the clean paper towel, indicating that bullfrogs did not respond to chemical cues from the predator snake. In the snake tongue flick experiments, snakes displayed no difference in TFS and TS toward rice frogs and bullfrogs but displayed more TFS and TS toward rice frogs and bullfrogs than toward toads and controls, suggesting that rice frogs and bullfrogs may share similar chemical cues, which could be identified as prey by the snakes. Moreover, the Jacobs' index of bullfrogs was above a mean of 0 in artificial ponds with (treatment C) and without a control for body size (treatment B) and in bullfrog-invaded water bodies, indicating that bullfrogs were the preferred prey for the snakes. Furthermore, snakes preferred bullfrogs over three native species in artificial pond experiments and over all four native species in the invaded sites. These results suggest that bullfrogs were subjected to higher predation pressures from the snakes than were native species. There was a positive correlation between the proportion of bullfrogs in snake diets and the density of bullfrogs in PSWDs. Our results are consistent with the predictions of the exotic prey naïveté hypothesis, but provide no support for the native enemy naïveté hypothesis.

Bullfrog tadpoles are known to discriminate and respond differently to different native predators in their native range [69], based on the palatability of the bullfrog tadpoles to the predators. The tadpoles can also recognize chemical cues of predators that may find them palatable in their invaded range of Willamette Valley, Oregon in the United States [40]. Surprisingly, we found that bullfrogs were not able to detect or respond to the cues from red-banded snakes. The naïveté of bullfrogs to the snakes is unlikely to arise from ontogenetic naiveté – lack of exposure to the snakes during development. Animals with ontogenetic naïveté often keep some limited innate anti-predator defenses [8], [70] or can learn to recognize previously unfamiliar predators by detecting conspecific alarm cues [71], [72], [73]. Effective antipredator responses can be quickly re-built after exposure to a predator. All bullfrogs in these experiments were collected from water bodies where the snakes were found. Because bullfrogs have been in water bodies in Daishan for over 20 years [55], it is likely that they had previously encountered the snakes or associated them with damaged conspecifics. The naiveté of bullfrogs most likely comes from evolutionary naiveté – no evolutionary history with the snakes. Although bullfrogs in their native range are the prey of some native snakes [74], [75], the red-banded snake may be a novel predator on the bullfrogs in bullfrog-invaded sites on Daishan Island. All of the species in the genus *Dinodon* are distributed only in South and East Asia [20], [21]. Bullfrogs have no co-evolutionary history with any snakes of the genus *Dinodon*, and are unlikely to have experienced selection by these snakes, including red banded snakes in the bullfrogs' native range. One explanation for their failure to recognize the chemical cues from red-banded snakes is that there might be little cue similarity between the red-banded snake and native snakes from the bullfrogs' native range [6]. Thus, bullfrogs may not identify the cues of the red-banded snake as a predation risk. An alternative explanation is that bullfrogs detect the cue but see it as one with no predation risk. Bullfrogs have been shown to prey upon smaller snakes in their native range [76], [77]. They might misinterpret cues of red-banded snakes as potential prey cues because they lack a shared evolutionary history with the red-banded snake. Such naiveté resulting from the misinterpretation by bullfrogs would hinder any defensive behaviors toward the snakes.

In comparison, native anuran species showed some effective antipredator responses to red-banded snakes. Both rice frogs and pond frogs spent more time on the clean paper towel side in the chemical cue avoidance experiment, indicating they could use chemical cues from the snakes to avoid predation. This avoidance behavior affords two species some defense against the snakes [36], [78]. Consistent with studies on other toad species [36], [38], toads showed no avoidance response to the chemical cues of the red-banded snake. This lack of avoidance arises because the toads have low vulnerability to predation by the snakes [79], [80]. Species in Bufonidae generally have noxious or toxic granular glands as antipredator defenses [81], [82], which render them unpalatable to predators including snakes. Red-banded snakes showed lower TFS and TS toward toads than pond frogs, rice frogs, or bullfrogs and had the lowest Jacobs'index for toads in the laboratory experiments and in the wild, suggesting that the toads are unpalatable to the snakes. We did not perform recognition experiments and diet preference experiments in artificial ponds for Japanese frogs due to the difficulty of collecting an adequate sample size from the wild. However, the Jacobs'index of Japanese frogs was <0 in non-invaded sites and in bullfrog-invaded water bodies. Moreover, the Jacobs'index of Japanese frogs was lower than that of bullfrogs. These results suggest that Japanese frogs might have effective defenses against red-banded snakes and experience lower predation pressures than bullfrogs.

Higher predation pressures of red banded snake on bullfrogs than native anurans on Daishan Island was consistent with interactions among ‘meat ant’, invasive cane toads and native anurans in Australia [18]. Cane toad metamorphs were more susceptible to predation by native enemy ‘meat ants’ than were seven native anuran species due to the cane toads' ineffective defense response when attacked. Our results, combined with the observation in Australia, suggest that exotic species may be readily attacked by native enemies in the exotic species' new range. Such heavier predation of native enemies on exotic species might prevent or constrain the establishment or spread of the exotic species.

Many studies have reported that native enemies can change their food habits by incorporating a larger proportion of exotic prey into their diet [83], [84], [85]. The mechanisms behind these observations remain unexplored [7], [86]. Our results suggest that red-banded snakes have altered their diet by consuming mostly bullfrogs and preferring bullfrogs over native anuran species. This preference is likely because bullfrogs were naïve to the snakes, whereas native anuran species have effective anti-predator defenses. As a consequence, the preference of the snakes for bullfrogs may contribute to the positive correlations between proportion of bullfrogs in snake diet and the density of bullfrogs in water bodies. As rice frogs are the preferred prey for snakes in non-bullfrog invaded sites, and are the second most common food item by number (the third by wet mass) after bullfrogs in bullfrog-invaded sites, their abundances may influence the snake diet.

The ERH assumes that the success of invasive species in a new range is partly due to their release from native enemies and partly because native enemies prefer native prey [6], [11], suppressing competition of native prey with the exotic species. Red-banded snakes did not prefer native species, preferring bullfrogs over native anuran species in invaded sites and providing no support for this hypothesis. These results are partly consistent with the predictions of the ISH [6], [11], which posits that exotic species are subjected to higher predation risk from native enemies than native prey species. Higher predation pressure from red-banded snakes may increase biotic resistance to bullfrog invasions. This result is in accordance with the predictions of the biotic resistance hypothesis [2].

In our study area, bullfrogs had high population numbers in ponds where there was also a high occurrence of red-banded snakes. This raises a basic question: why do red-banded snake not prevent bullfrog invasions in water bodies inhabited by the snakes? It may be that the naiveté of bullfrogs to the snakes is not the only factor that ultimately determines the establishment of bullfrogs. Other factors, such as propagule pressure and human hunting pressures on bullfrogs have been found to be related to the successful establishment of bullfrog populations in a water body [55], [87], [88], [89]. Bullfrogs may also enjoy a novelty advantage associated with the naiveté of native prey to bullfrogs and enemies other than the red-banded snake, which may offset the effects of the snakes. These hypotheses remain untested.

Our results provide strong evidence for the exotic prey naiveté hypothesis and the ‘increased susceptibility’ hypothesis in predator-prey systems. The results confirm that native enemies to which exotic species are naive exert biological resistance to the exotic species. Once established and causing impacts, the complete removal of invasive species can be extremely costly, deleterious, and often impossible [6], [7], [8]. Traditional biocontrol of pests by introducing alien enemies or parasites often brings the problem of biological invasions [3], [11]. Using native enemies to which invasive species are naïve may avoid this problem and can be an effective approach to managing the invasive species [90]. It is important to identify the enemies to which invasive prey are naïve, based on reciprocal recognition between exotic prey and native enemies and the diet preferences of native enemies. As invasive species are naïve to the native enemies, whereas native prey are not, the increase in abundance of the native enemies with human assistance should help to reduce or even extirpate established populations of invasive prey[90]. Many native enemies have been threatened by habitat destruction, overexploitation and other factors [7]. There is a need to protect native enemies from these threats to provide biotic resistance to the exotic prey invasions.

## Supporting Information

Supporting Information S1 Location of study site in the Zhoushan archipelago, Zhejiang province, China.
(DOC)

Supporting Information S2 Body sizes (SVL,mm) of native enemy and anuran species in the study system on Daishan, China. See references [1], [2].
(DOC)

Supporting Information S3 The capture and maintenance of snakes and anurans.
(DOC)

Supporting Information S4 Diagram represents the testing terrarium used for the investigation on the prey preference of adult red banded snakes.
(DOC)

Supporting Information S5 Artificial ponds used for the diet preference experiments of red banded snake for bullfrog vs three native anuran species. The top picture shows one pond. The bottom picture shows that the ponds covered with a sunshade screen. The ponds are similar size and in two rows.
(DOC)

Supporting Information S6 The average snout-vent length (mm) and standard deviation (number of individuals) of animals used in treatments of artificial pond experiments. Each treat has been performed in triplicates. All animals were measured at the beginning of the experiments. Treaxent A represents the snake predation on three native anurans. Treatment B represents snake predation on random size anurans. C represents snake predation on similar size anurans. There is no difference in snout-vent length (Ln transformed) for red banded snake among treatments (One-way ANOVA, *F* = 0.643, *df* = 2, *p* = 0.558), and no difference in snout-vent length (Ln transformed) among anuran species in treatment C (*F* = 0.785, *df* = 3, 20, *p* = 0.516). See text.
(DOC)
