# Supplementary material for: An Exotic Species Is the Favorite Prey of a Native Enemy
Source: PLoS One. 2011 Sep 6;6(9):e24299. doi: 10.1371/journal.pone.0024299 (PMC3167836; doi:10.1371/journal.pone.0024299)
Supplement: Supporting Information S1 — Location of study site in the Zhoushan archipelago, Zhejiang province, China. (DOC) [file pone.0024299.s001.doc]

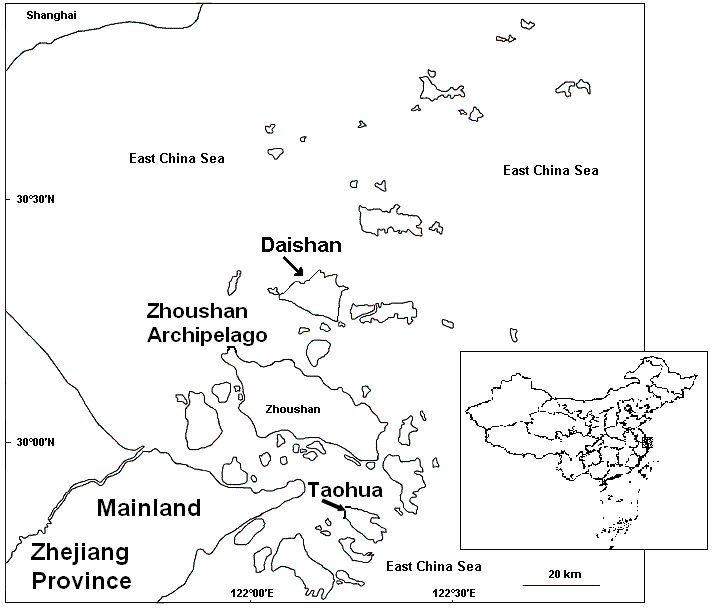


Supporting information S1. Location of study site in the Zhoushan archipelago, Zhejiang province, China.
