# Supplementary material for: An Exotic Species Is the Favorite Prey of a Native Enemy
Source: PLoS One. 2011 Sep 6;6(9):e24299. doi: 10.1371/journal.pone.0024299 (PMC3167836; doi:10.1371/journal.pone.0024299)
Supplement: Supporting Information S2 — Body sizes (SVL,mm) of native enemy and anuran species in the study system on Daishan, China. See references [1], [2]. (DOC) [file pone.0024299.s002.doc]

Supporting information S2. Body sizes (SVL,mm) of native enemy and anuran species in the study system on Daishan, China. See references [1,2]

| Species | Males | Females |
| --- | --- | --- |
| Red banded snake (*Dinodon Rufozonatum*) | 560-1040 | 555-1130 |
| Bullfrog (*Lithobates [Rana] catesbeianus*) | 85.6-157.1 | 93.6-162.6 |
| Pond frog (*Rana nigromaculata*) | 49-70 | 55-90 |
| Rice frog (*Fejervarya[Rana]limnocharis*) | 33-39 | 40-50 |
| Japanese frog (*R.japonica*) | 40.4-53.6 | 41.2-67.5 |
| Toad (*Bufo bufo*) | 55-110 | 65-126 |

1. Huang M, Jin Y, Cai C (1990) Fauna of Zhejiang: Amphibia, Reptilia: Zhejiang Science and Technology Publishing House.

2. Xuan L, Yiming L, McGarrity M (2010) Geographical variation in body size and sexual size dimorphism of introduced American bullfrogs in southwestern China. Biological Invasions 12: 2037-2047.
