# Supplementary material for: An Exotic Species Is the Favorite Prey of a Native Enemy
Source: PLoS One. 2011 Sep 6;6(9):e24299. doi: 10.1371/journal.pone.0024299 (PMC3167836; doi:10.1371/journal.pone.0024299)
Supplement: Supporting Information S3 — The capture and maintenance of snakes and anurans. (DOC) [file pone.0024299.s003.doc]

Supporting information S3. The capture and maintenance of snakes and anurans

Daily air temperature during capture ranged from 15 – 30 °C. Snakes were temporarily stored in ventilated sacks and brought to the laboratory where they were individually housed in covered plastic boxes (50 cm × 40 cm × 30 cm) in which a dish was provided with water *ad libitum*. Because prey avoidance can be accentuated when conspecifics are fed to predators, we fed snakes with carp (Cyprinus spp.), which are a neutral diet that could not bias interspecific comparisons of prey responses [1,2]. Each snake was fed 3-5 carp (about 60 g/carp) every 3 days until 5 days before the trials began [1,2]. Before the experiments started, we recorded the snout–vent length (SVL) of each snake to the nearest 0.2 cm and body mass to the nearest 0.1 g. Each snake was used only once in the snake tongue flicking experiment and/or once in the chemical cue avoidance reaction experiment [3].

All frogs and toads were captured by dip-net or by hand at night (1900–2400 h) with the help of a 12-volt DC lamp. Captured anurans were temporarily kept in nylon bags and brought to the laboratory. They were kept in plastic tanks with only conspecifics and were fed crickets (Gryllus spp.) *ad libitum* until 48 h before trials. We recorded their SVL to the nearest 0.2 cm and body mass was recorded to the nearest 0.1 g before the experiments. In order to avoid disturbing male breeding behaviors during the breeding season, we only used female anurans in the chemical cue avoidance experiments [3,4,5]. Individual frogs and toads were not used in more than one trial unless otherwise stated.

All animals were maintained on a 12:12 hour (light:dark) photoperiod at approximately 20 °C. All anurans were kept in a separate room from the snakes so as not to introduce mixed odors to the experimental room or acclimate the snakes to ambient odors.

1. Murray D, Roth J, Wirsing A (2004) Predation risk avoidance by terrestrial amphibians: the role of prey experience and vulnerability to native and exotic predators. Ethology 110: 635-647.

2. Aubret F, Burghardt GM, Maumelat S, Bonnet X, Bradshaw D (2006) Feeding preferences in 2 disjunct populations of tiger snakes, Notechis scutatus (Elapidae). Behavioral Ecology 17: 716-725.

3. Downes SJ (2002) Size-dependent predation by snakes: selective foraging or differential prey vulnerability? Behavioral Ecology 13: 551-560.

4. Howard RD (1978) The Evolution of Mating Strategies in Bullfrogs, Rana catesbeiana. Evolution 32: 850-871.

5. Judge KA, Brooks RJ (2001) Chorus participation by male bullfrogs, Rana catesbeiana: a test of the energetic constraint hypothesis. Animal Behaviour 62: 849-861.
