# Supplementary material for: An Exotic Species Is the Favorite Prey of a Native Enemy
Source: PLoS One. 2011 Sep 6;6(9):e24299. doi: 10.1371/journal.pone.0024299 (PMC3167836; doi:10.1371/journal.pone.0024299)

Supporting information S4. Diagram represents the testing terrarium used for the investigation on the prey preference of adult red banded snakes.


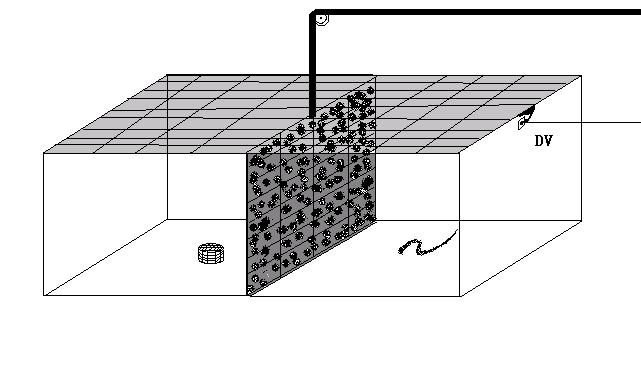

Supplement: Supporting Information S4 — Diagram represents the testing terrarium used for the investigation on the prey preference of adult red banded snakes. (DOC) [file pone.0024299.s004.doc]
