# Supplementary material for: An Exotic Species Is the Favorite Prey of a Native Enemy
Source: PLoS One. 2011 Sep 6;6(9):e24299. doi: 10.1371/journal.pone.0024299 (PMC3167836; doi:10.1371/journal.pone.0024299)
Supplement: Supporting Information S5 — Artificial ponds used for the diet preference experiments of red banded snake for bullfrog vs three native anuran species. The top picture shows one pond. The bottom picture shows that the ponds covered with a sunshade screen. The ponds are similar size and in two rows. (DOC) [file pone.0024299.s005.doc]

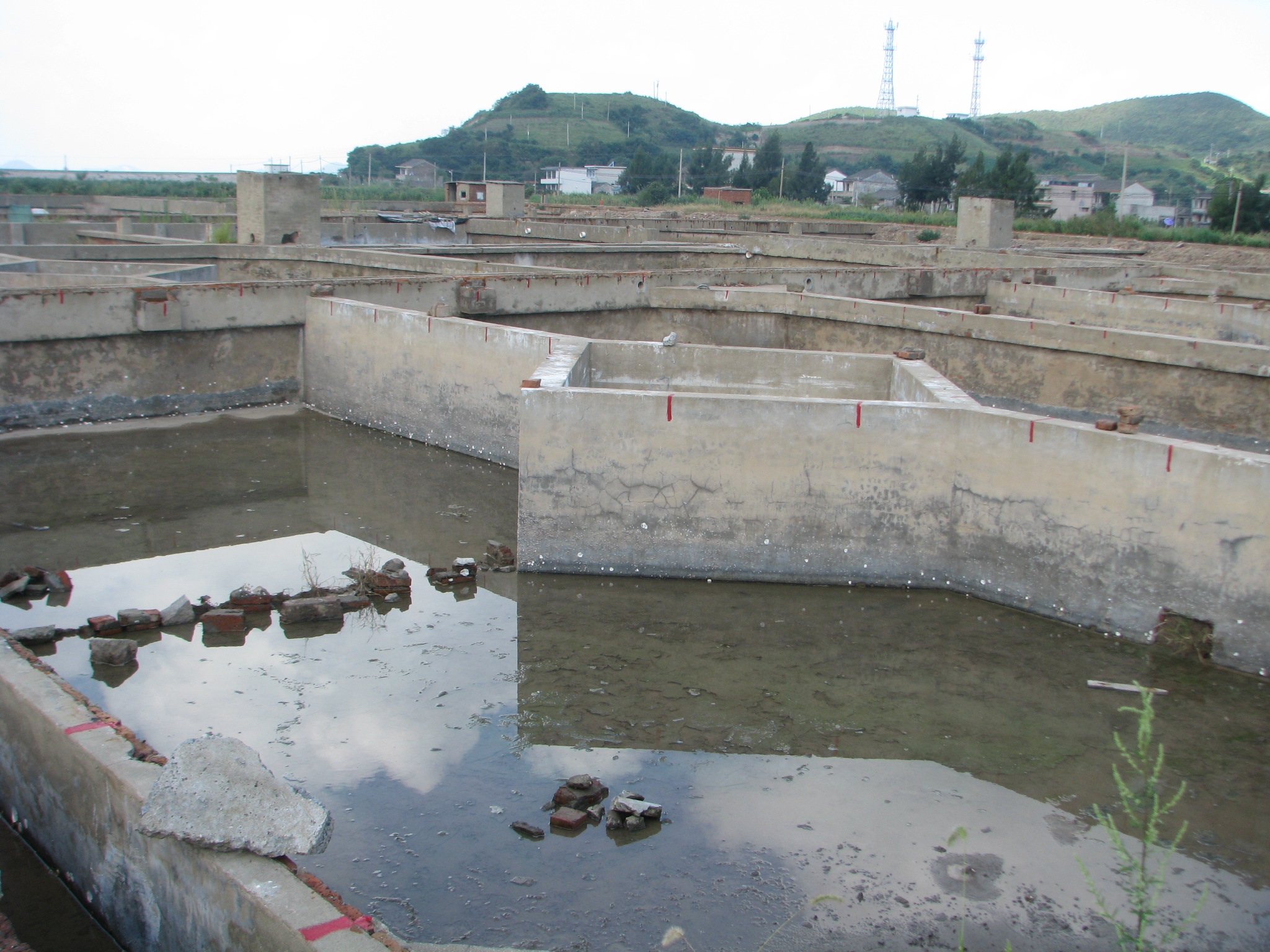


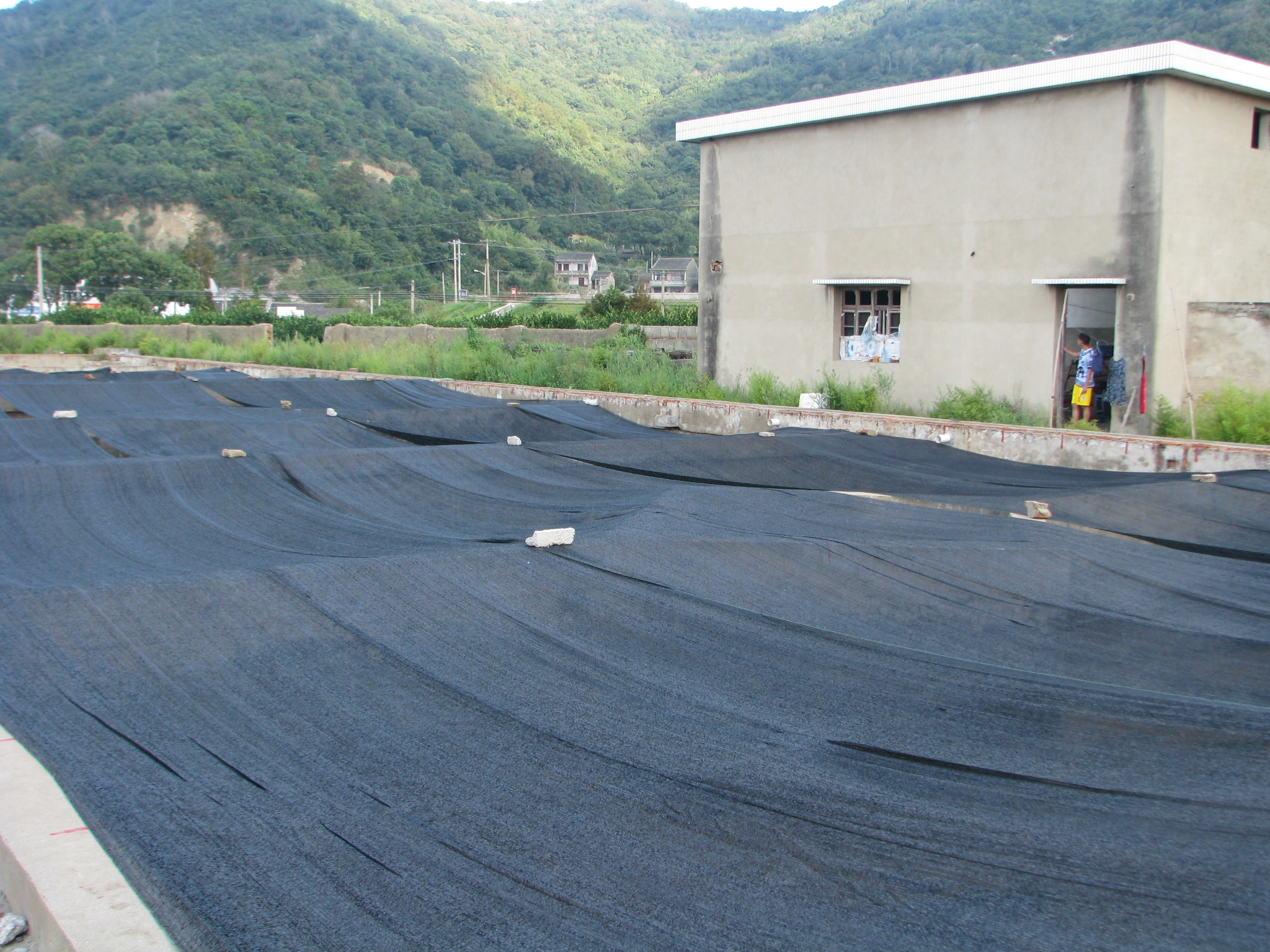


Supporting information S5. Artificial ponds used for the diet preference experiments of red banded snake for bullfrog vs three native anuran species. The top picture shows one pond. The bottom picture shows that the ponds covered with a sunshade screen. The ponds are similar size and in two rows.
