# Supplementary material for: An Exotic Species Is the Favorite Prey of a Native Enemy
Source: PLoS One. 2011 Sep 6;6(9):e24299. doi: 10.1371/journal.pone.0024299 (PMC3167836; doi:10.1371/journal.pone.0024299)
Supplement: Supporting Information S6 — The average snout-vent length (mm) and standard deviation (number of individuals) of animals used in treatments of artificial pond experiments. Each treat has been performed in triplicates. All animals were measured at the beginning of the experiments. Treaxent A represents the snake predation on three native anurans. Treatment B represents snake predation on random size anurans. C represents snake predation on similar size anurans. There is no difference in snout-vent length (Ln transformed) for red banded snake among treatments (One-way ANOVA, F = 0.643, df = 2, p = 0.558), and no difference in snout-vent length (Ln transformed) among anuran species in treatment C (F = 0.785, df = 3, 20, p = 0.516). See text. (DOC) [file pone.0024299.s006.doc]

Supporting information S6. The average snout-vent length (mm) and standard deviation (number of individuals) of animals used in treatments of artificial pond experiments. Each treat has been performed in triplicates. All animals were measured at the beginning of the experiments. Treatment A represents the snake predation on three native anurans. Treatment B represents snake predation on random size anurans. C represents snake predation on similar size anurans. There is no difference in snout-vent length (Ln transformed) for red banded snake among treatments (One-way ANOVA, *F* =0.643, *df* = 2, *p* = 0.558), and no difference in snout-vent length (Ln transformed) among anuran species in treatment C (*F* = 0.785, *df* = 3, 20, *p* = 0.516). See text.

| Treatment | *Red banded snake*  *Dinodon rufozonatum* | *Bullfrog*  *Rana catesbeiana =Lithobates catesbeianus* | *Pond frog*  *Rana nigromaculata* | *Rice frog*  *Fejervarya (Rana) limnocharis* | *Toad*  *Bofu bufo* |
| --- | --- | --- | --- | --- | --- |
| A | 108.5 ± 5.9 (3) | --- | 6.97 ± 0.63 (6) | 4.72 ± 0.77 (6) | 6.80 ± 0.61 (6) |
| B | 104.5 ± 11.3 (3) | 8.56 ± 2.30 (6) | 6.65 ± 0.69 (6) | 4.63 ± 0.29 (6) | 7.16 ± 0.65 (6) |
| C | 111.8 ± 5.0 (3) | 5.64 ± 0.22 (6) | 5.64 ± 0.20 (6) | 5.52 ± 0.33 (6) | 5.47 ± 0.15 (6) |
